# Precardiac organoids form two heart fields via Bmp/Wnt signaling

**DOI:** 10.1038/s41467-018-05604-8

**Published:** 2018-08-07

**Authors:** Peter Andersen, Emmanouil Tampakakis, Dennisse V. Jimenez, Suraj Kannan, Matthew Miyamoto, Hye Kyung Shin, Amir Saberi, Sean Murphy, Edrick Sulistio, Stephen P. Chelko, Chulan Kwon

**Affiliations:** 1 Division of Cardiology, Department of Medicine, Johns Hopkins University School of Medicine, Baltimore, MD 21205 USA; 2 Institute for Cell Engineering, Johns Hopkins University School of Medicine, Baltimore, MD 21205 USA; 3 Cellular and Molecular Medicine, Johns Hopkins University School of Medicine, Baltimore, MD 21205 USA

## Abstract

The discovery of the first heart field (FHF) and the second heart field (SHF) led us to understand how cardiac lineages and structures arise during development. However, it remains unknown how they are specified. Here, we generate precardiac spheroids with pluripotent stem cells (PSCs) harboring GFP/RFP reporters under the control of FHF/SHF markers, respectively. GFP^+^ cells and RFP^+^ cells appear from two distinct areas and develop in a complementary fashion. Transcriptome analysis shows a high degree of similarities with embryonic FHF/SHF cells. Bmp and Wnt are among the most differentially regulated pathways, and gain- and loss-of-function studies reveal that Bmp specifies GFP^+^ cells and RFP^+^ cells via the Bmp/Smad pathway and Wnt signaling, respectively. FHF/SHF cells can be isolated without reporters by the surface protein Cxcr4. This study provides novel insights into understanding the specification of two cardiac origins, which can be leveraged for PSC-based modeling of heart field/chamber-specific disease.

## Abstract (web summary)

The heart arises from distinct progenitor cells of both the first and second heart fields (FHF and SHF). Here, the authors generated precardiac organoids from mouse and human pluripotent cells and show that FHF and SHF cells form similarly to their in vivo counterparts in response to BMP and Wnt signalling, respectively.

## Introduction

Recent advances in cardiac developmental biology have led us to learn how diverse lineages and different anatomical structures of the heart arise from the two sets of molecularly distinct cardiac progenitor cells (CPCs), referred to as the first and second heart field (FHF and SHF). However, it remains unclear how the FHF and SHF populations are specified from mesodermal progenitors and which factors and mechanisms regulate their induction.

In early developing embryos, proper interactions of morphogens, including bone morphogenetic proteins (Bmps), Wnts, fibroblast growth factors, activin/nodal, play critical roles in formation of the primitive streak, progression of gastrulation and mesodermal patterning in the anterior–posterior axis^1–5^. While numerous loss- and gain-of-function studies have demonstrated the importance of these pathways in early heart development, their precise roles in heart field induction and allocation remain to be determined^6^. However, recent studies provided evidence that heart field progenitors are assigned to a specific developmental path from nascent mesoderm marked by basic-helix-loop-helix (bHLH) transcription factor Mesp1 during gastrulation^7,8^, suggesting that the specification occurs soon after formation of three germ layers. Several transcription factors are known to have essential roles for precardiac mesoderm development^9,10^: the T-box transcription factor Eomesodermin and the bHLH Id family of genes promote formation of cardiovascular mesoderm by activating Mesp1 during gastrulation, which in turn regulates expression of genes belonging to the cardiac transcriptional machinery such as Hand2, Gata4, Nkx2.5, and Myocd^11–13^. Retrospective lineage analyses revealed that Mesp1^+^ cells contribute to both heart fields^14^. The FHF, comprising the cardiac crescent, is identified by expression of Hcn4 and Tbx5^15,16^, before giving rise to the left ventricle (LV) and part of the atria, whereas the SHF is marked by transient expression of Tbx1, Fgf8/10, Isl1, and Six2, and exclusively contributes to the outflow tract (OT), the right ventricle (RV) and part of the atria^17–22^. SHF cells are multipotent CPCs that can be fated to various cardiac cell types, such as cardiomyocytes, smooth muscle cells, endothelial cells, and fibroblast cells, while FHF cells mostly become cardiomyocytes^8,15^.

With the capability to differentiate into any type of body cell, pluripotent stem cells (PSCs) have emerged as a powerful tool to study development and disease^23–25^. Particularly, the development of human-induced PSCs (iPSC) technology and robust cardiac differentiation protocols^26^ has enabled the study of disease-causing cellular and molecular events that manifest in congenital heart defects (CHDs), the most common birth defect and birth-related deaths in humans. Both genetic and environmental influences have been implicated to cause disruption of the normal series of morphogenetic embryonic developmental events that affects the occurrence of heart abnormalities. CHDs are often restricted to regions of the heart arising from the FHF or SHF^27,28^ and/or linked to mutations of genes that regulate development of the individual heart fields^16,17,19,29^. This raises the question whether chamber-specific heart abnormalities originate from abnormal heart field development. Additionally, efforts in tissue engineering and three-dimensional (3D) bioprinting are now focused on developing heart chamber-specific models and to generate chamber-specific heart tissue from hiPSCs to replace damaged heart muscle^30^. Yet, it remains unknown whether the distinct heart field populations can be generated in a PSC system.

In the present study, we generated 3D precardiac spheroids with PSCs that allows induction of FHF/SHF progenitors sharing a high degree of similarities with their in vivo counterparts. We further demonstrate how Bmp and Wnt/β-catenin signaling control the specification of FHF and SHF progenitors in mouse and human PSCs, enabling selective induction of FHF or SHF cells. The heart field progenitors can be identified and isolated without transgene reporters by the cell surface protein Cxcr4 for PSC-based modeling of CHDs.

## Results

### FHF/SHF-like cells are induced in spheroid PSC culture

**Fig. 1:**
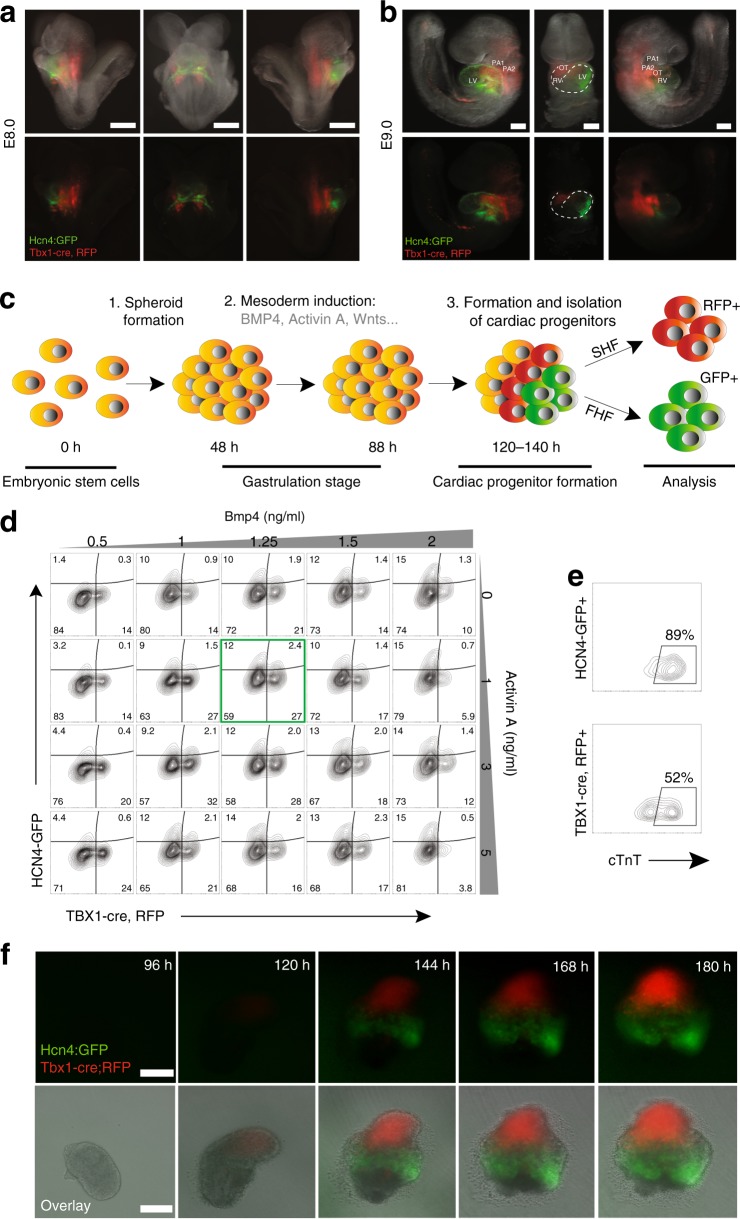
FHF/SHF-like cells are induced in spheroid PSC culture. **a** Live imaging of Hcn4-GFP, Tbx1-Cre, Ai9 mice E8.0. GFP is exclusively expressed in the cardiac crescent and primitive myotube, whereas RFP is expressed in the posterior region. **b** Live imaging of Hcn4-GFP, Tbx1-Cre, Ai9 mice E9.0. GFP expression is restricted to the LV and atria whereas RFP is expressed in the pharyngeal region posterior to the heart, the outflow tract and RV. **c** Schematic of the strategy used to generate and differentiate ESC-derived cardiac spheroids. **d** Flow cytometric analyses of Hcn4-GFP^+^ and Tbx1-Cre, RFP^+^ in cardiac spheroids after 5.5 days of differentiation. **e** Flow cytometric analyses of GFP^+^/cTnT^+^ and RFP^+^/cTnT^+^ cells at day 9 in cardiac spheroids. **f** Representative images from live imaging time-lapse experiments of a differentiating cardiac spheroid. White scale bars indicate 100 μm

Lineage tracing experiments with CPC markers, including Hcn4, Tbx5, Isl1, and Tbx1, have identified distinct FHF and SHF structures in developing mouse embryos. To verify if these markers faithfully label the FHF or the SHF, we examined their expression in mice between embryonic days 7.5 and 9.5 post fertilization (E7.5 and E9.5). Hcn4 and Tbx5 were both expressed in the FHF (Supplementary Fig. 1a–d), and Tbx1 was expressed in the SHF and structures derived thereof (Supplementary Fig. 1a–c, i). When traced with *Isl1*^*Cre*^ mice^31^, cells expressing Isl1, regarded as a SHF marker, gave rise to both FHF and SHF structures (Supplementary Fig. 1e–h), including the entire LV at E9.5. Isl1 lineage tracing further revealed that Nkx2.5-expressing cells in the cardiac crescent are derived from Isl1^+^ cells (Supplementary Fig. 1e, f). This suggests that Isl1 marks undifferentiated CPCs of both heart fields. Based on these analyses, we generated mice expressing green/red fluorescent protein (GFP/RFP) in FHF cells/SHF lineage cells by crossing *Tbx1*^*Cre*^; *Ai9* mice with *Hcn4*^*GFP*^ mice^17,32^. In this system, GFP is expressed in Hcn4^+^ cells in the FHF^15^ and RFP permanently marks Tbx1 progeny in the SHF^17^. As expected, GFP was expressed in the cardiac crescent, whereas RFP labeled the region dorsal to the crescent where SHF cells are located (Fig. 1a). At E9.0, GFP was expressed in the LV, and RFP was restricted to the pharyngeal mesoderm and the OT/RV (Fig. 1b and Supplementary Fig. 1i), confirming that GFP and RFP mark FHF cells and SHF cells, respectively.

We next established an embryonic stem cell (ESC) line (ESC^*Hcn4-GFP; Tbx1-Cre; Ai9*^) from the mice to determine if heart field specification can be recapitulated in a PSC system. We hypothesized that a 3D multicellular system would better resemble heart field development in vivo, as early development is a highly dynamic process that involves tissue–tissue interactions between multiple cell types. Since CPCs are specified during mid-late gastrulation^7,8^, we generated multicellular 3D spheroids with the PSCs and treated them with various concentrations of Activin A and Bmp4 to determine whether induction of the early mesoderm influences heart field specification (Fig. 1c, d). After 5 days of differentiation (120 h), GFP^+^ and RFP^+^ cells started to appear in the spheroids. We analyzed the spheroids for GFP^+^ and RFP^+^ cells by fluorescent activated cell sorting (FACS) at day 5.5 (132 h). The FACS data showed that cells are either GFP^+^ or RFP^+^ with <4% double positive (GFP^+^/RFP^+^) cells among all fluorescent cells (Fig. 1d), indicating that FHF and SHF cells are distinctively specified in cardiac spheroids. The double positive GFP^+^/RFP^+^ cells correlated with the total number of GFP^+^ and RFP^+^ cells (Supplementary Fig. 1j) and were sporadically interspersed within the GFP^+^ domain (Supplementary Fig. 1k), implying that these rare events may be in vitro artifacts. Thus, we excluded these cells from further analysis.

We examined the overall effects on cardiogenesis by analyzing the number of cardiomyocytes at day 9 (Supplementary Fig. 1). Varying Bmp4 concentrations had a profound effect on cardiogenesis with the number of cTnT^+^ cardiomyocytes reaching >30% with 1.25 ng/mL Bmp4, more than a 10-fold difference compared to 0.5 ng/mL Bmp4, whereas increasing Activin A levels had a modest effect on cardiogenesis compared to control (Supplementary Fig. 1m, n), implying an important role for Bmp signaling during early cardiogenesis. To determine the individual cardiomyogenic potential of GFP^+^ and RFP^+^ cells, we analyzed their cardiomyocyte contribution from the most cardiogenic condition (1.25 ng/mL Bmp4, 1 ng/mL Activin A) at day 9. 89% of GFP^+^ cells were positive for cTnT, showing a differentiation bias toward a cardiomyogenic cell fate, whereas 52% of RFP^+^ cells were positive for cTnT (Fig. 1d). This indicates that GFP^+^ cells are primarily unipotent and cardiomyogenic, whereas RFP^+^ cells likely give rise to several cardiac cell lineages.

To monitor the process of GFP^+^/RFP^+^ cell induction, we performed a time-lapse analysis of the spheroids (Fig. 1e, Supplementary Movie 1). At 120 h (5 days) of differentiation, areas of GFP^+^ and RFP^+^ cells started to appear adjacent to each other. GFP^+^ zones generally appeared in the periphery of cardiac spheroids, whereas RFP^+^ zones appeared more central (Supplementary Fig. 1k). After 168 h (7 days), the majority of cardiomyocytes (cTnT^+^ cells) in the cardiac spheroids were GFP^+^, whereas RFP^+^ cardiomyocytes continuously increased between 168–204 h (7–9 days) (Supplementary Fig. 1o), demonstrating that cardiomyogenesis is delayed in RFP^+^ CPCs compared to GFP^+^ CPCs, similar to in vivo, where the SHF does not contribute to the myocardium until the looping stage (E8.5)^33^. It is worth noting that both populations maintained the complementary pattern over time within the spheroids (Fig. 1e), analogous to developing heart fields in vivo.

### PSC-derived FHF/SHF progenitors are similar to endogenous FHF/SHF progenitors in gene expression and differentiation potential

Next, we sought to determine the cellular identities of PSC-derived GFP^+^ and RFP^+^ cells. To do this, GFP^+^ and RFP^+^ CPCs were FACS-isolated from the spheroids at day 5.5 or from *Hcn4*^*GFP*^; *Tbx1*^*Cre*^; *Ai9* mouse embryos at E7.75 and subjected to RNA-sequencing. Genome-wide transcriptome analysis revealed a high correlation between in vivo and in vitro CPCs (GFP: in vitro vs. in vivo, *R*^2^ = 0.91, RFP: in vitro vs. in vivo, *R*^2^ = 0.98) (Supplementary Fig. 2a), indicating similar gene expression profiles between PSC-derived cells and their in vivo counterparts. Expression levels of Hcn4 and Tbx1 were also confirmed in the analyzed populations (Supplementary Fig. 2b).

**Fig. 2:**
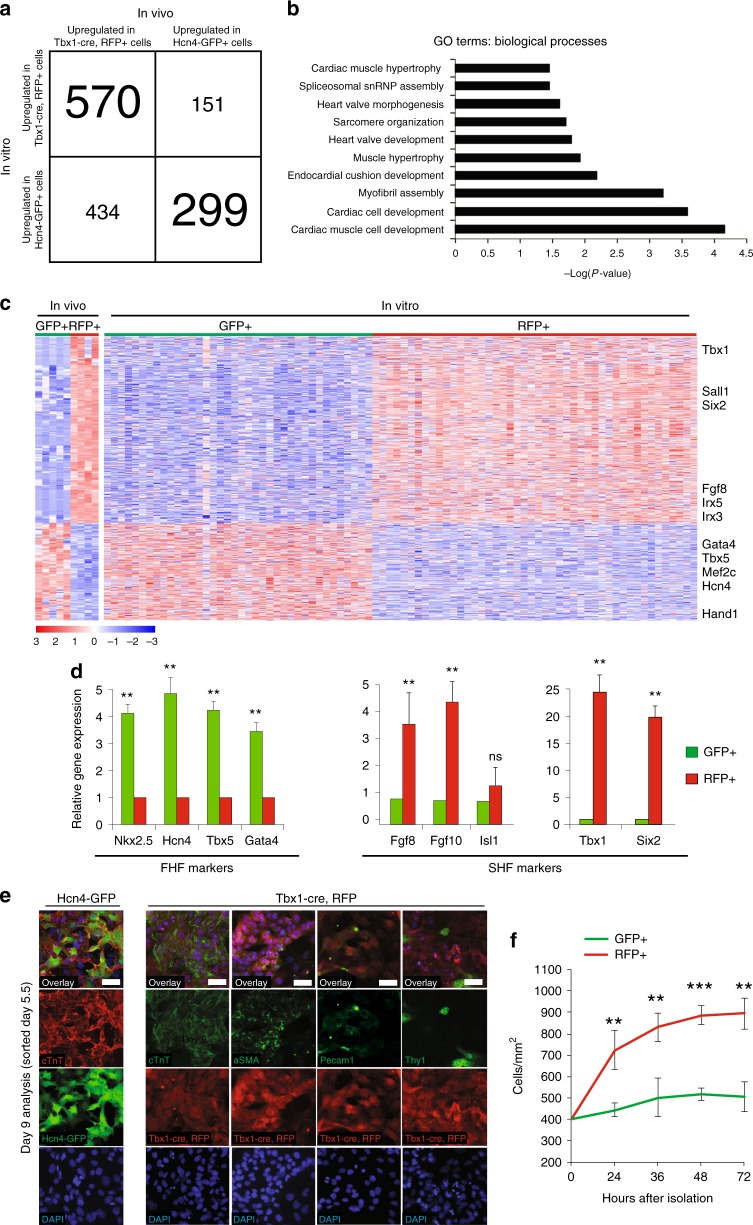
PSC-derived FHF/SHF cells are similar to FHF/SHF cells in embryos. **a** RNA-seq analysis of differentially regulated genes between Hcn4-GFP^+^ and Tbx1-Cre, RFP^+^ CPCs in vivo and in vitro. The DESeq2 package identified 1454 genes that were differentially regulated between Hcn4-GFP^+^ and Tbx1-cre, RFP^+^ CPCs in vivo and in vitro (Benjamini–Hochberg adjusted *p*-value < 0.1). Upregulation in the GFP^+^ or RFP^+^ CPCs was determined using the directionality of fold change from DESeq2. 869 genes showed upregulation in the same CPC population both in vivo and in vitro. **b** Gene Ontology (GO) term analysis of 869 genes identified in **a**. Top ten biological processes enriched in the gene list (Bonferroni adjusted *p*-values < 0.05) are shown. **c** RNA-seq heatmaps of CPCs both in vivo and in vitro using the 869 genes identified in **a**. Heatmaps show row-scaled regularized logarithmic transformation of counts as produced by the DESeq2 package. Hcn4-GFP^+^ and Tbx1-Cre, RFP^+^ CPCs cluster separately based on expression patterns of these genes both in vivo and in vitro. Select known FHF and SHF markers are labeled. **d** qPCR analyses of selected genes involved in early CPC development of PSC-derived Hcn4-GFP CPCs and Tbx1-Cre, RFP isolated day 5.5. Data are mean ± SEM; **p < 0.01; ns, not significant (*p* > 0.05). *p* values were determined using a paired Student’s *t* test. **e** Immunohistochemistry analyses of cTnT, Pecam-1, Tny1, and aSMA at PSC-derived Hcn4-GFP CPCs and Tbx1-Cre, RFP CPCs isolated day 5.5 and analyzed day 9. White scale bars indicate 50 μm. **f** Cell counts of Hcn4-GFP^+^ CPCs and Tbx1-Cre, RFP^+^ CPCs isolated day 5.5. All data are mean ± SEM; *n* = 3; **p < 0.01; ***p < 0.001. *p* values were determined using a paired Student’s *t* test

We identified 1968 genes that were differentially regulated between GFP^+^ and RFP^+^ cells in vivo (adjusted *p*-value < 0.1); of these, 1454 genes were differentially regulated between the GFP^+^ and RFP^+^ populations in vitro. Among these, 869 genes showed higher expression in the same population (i.e., GFP^+^ or RFP^+^) both in vitro and in vivo (Fig. 2a). Gene Ontology (GO) analysis for these genes showed enrichment for terms relevant to cardiovascular cellular and organ development (Fig. 2b, Supplementary Data 1). This gene list included known FHF genes (*Gata4, Tbx5, Mef2c, Hand1)* and SHF genes (*Sall1, Six2, Fgf8, Irx3, Irx5*) and could be used to distinguish GFP^+^ and RFP^+^ cells in vitro (Fig. 2c, Supplementary Data 1). 585 genes showed different expression patterns compared between in vitro and in vivo. GO analysis of these genes showed enrichment of terms such as ‘cell-substrate adhesion’ (Supplementary Fig. 2c, Supplementary Data 1), which is likely due to differences between the in vivo and in vitro microenvironments. The enrichment of FHF and SHF genes in GFP^+^ and RFP^+^ cells was further confirmed by qPCR analysis (Fig. 2d, Supplementary Data 2). *Isl1* was expressed in both cell types without significant difference in levels (Fig. 2d). This is consistent with our earlier finding (Supplementary Fig. 1e–h) and the previous reports that *Isl1* is a pan-cardiac marker^34,35^. Its expression is however downregulated at E8.5 in GFP^+^ cells (Supplementary Fig. 3), suggesting that *Isl1* is transiently expressed in the FHF. The prolonged expression of Isl1 in RFP^+^ cells may correlate with its required role for SHF development^21,36^.

To verify the cardiomyogenic potential of PSC-derived GFP^+^/RFP^+^ cells, we isolated the cells immediately after appearance of GFP and RFP (day 5.5), when no cTnT^+^ cells were detected, and differentiated for 4 days. Consistent with the earlier FACS analysis (Fig. 1d), GFP^+^ cells robustly gave rise to cardiomyocytes (Fig. 2e), suggesting that they are committed to a cardiomyogenic lineage. In vivo, SHF progenitors proliferate prior to differentiation and give rise to most cell types of the heart, including cardiomyocytes, the endothelium and fibroblasts (Supplementary Fig. 2d)^8,20,33,37^. Similarly, RFP^+^ cells gave rise to cells positive for cTnT (cardiomyocytes), Pecam-1 (endothelia), α-SMA (smooth muscle), and Thy1 (fibroblasts) (Fig. 2e). As Tbx1 is also expressed in head muscle progenitors in developing embryos^38^, we tested for the early muscle marker Myogenin in the spheroids at day 9. We did not detect meaningful percentages of RFP^+^ and Myogenin^+^ cells (0.26%) compared to RFP^+^ and cTnT^+^ cells (47%) (Supplementary Fig. 2e). In addition, cells positive for the epicardial marker Wilms tumor 1 (WT1) were nearly undetectable (0.062%) (Supplementary Fig. 2e), indicating that RFP^+^ almost exclusively give rise to cardiac lineages.

KEGG pathway analysis revealed increased cell cycle activity in RFP^+^ cells compared to GFP^+^ cells (Supplementary Fig. 2f). GFP^+^ cells showed increased activity of the p53 signaling pathway, commonly known as a negative regulator of cell cycle activity (Supplementary Fig. 2g). Consistently, RFP^+^ cells doubled in numbers within 36 h of culture, whereas GFP^+^ cells showed a modest level of proliferation (Fig. 2f). These indicate that RFP^+^ cells represent multipotent and proliferative CPCs analogous to the SHF in vivo. Since PSC-derived GFP^+^ and RFP^+^ represent distinct FHF and SHF CPCs, we tested their potential for heart field/chamber-specific disease modeling. To do this, we knocked down *Tbx5*, the causative gene for Holt-Oram syndrome, which is associated with left-sided ventricular heart malformation including hypoplastic left heart syndrome in humans and mice^39,40^. Reduced levels of Tbx5 significantly decreased the number of cardiomyocytes formed from GFP^+^ cells but had no effect on RFP^+^ cells (Supplementary Fig. 2h). Contrarily, knocking down *Tbx1*, a causative gene for DiGeorge syndrome associated with OT defects, negatively affected the proliferation of RFP^+^ cells, but not GFP^+^ cells (Supplementary Fig. 2i). Together, these findings support the recapitulation of the in vivo process and gene expression of GFP^+^ and RFP^+^ populations in the PSC spheroid system, and thus, this system may be used to model cellular and molecular heart field/chamber-specific events associated with CHDs.

### FHF and SHF progenitors are specified via the Bmp/Smad pathway and a Smad-independent Bmp/Wnt pathway, respectively, in PSC-derived spheroids

**Fig. 3:**
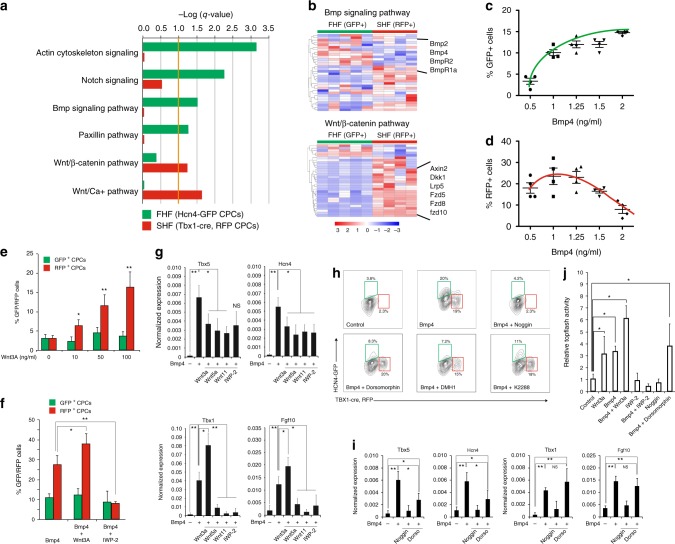
Bmp and Wnt activities regulate heart field specification in cardiac spheroids. **a** Ingenuity pathway analysis of genes differentially expressed in Hcn4-GFP^+^ and Tbx1-Cre, RFP^+^ CPCs in vivo. We focused our analysis on pathways involved with “organism growth and development”. Data are shown as logarithm of Benjamini–Hochberg adjusted p-value, with threshold for significance of *p*-value < 0.1. **b** RNA-seq heatmaps of selected differentially regulated genes (Benjamini–Hochberg adjusted *p*-value < 0.1) from Bmp signaling pathway and Wnt/β-catenin pathways. Data are shown as row-scaled regularized logarithmic transformation of counts as produced by the DESeq2 package. **c** Vertical scatter plot showing the effect of increasing Bmp4 on formation of Hcn4-GFP^+^ CPCs (green trend line). **d** Vertical scatter plot showing the effect of increasing Bmp4 on formation of Tbx1, RFP + CPCs (red trend line). Both analyses (**c**, **d**) were performed on GFP^+^/RFP^+^ percentages from flow cytometric analyses in Fig. 1d. **e** Number of Hcn4-GFP^+^ CPCs and Tbx1-Cre, RFP + CPCs after induction with increasing concentrations of Wnt3A. **f** Number of Hcn4-GFP^+^ CPCs and Tbx1-cre, RFP + CPCs after induction with Bmp4 (1.25 ng/mL) in combination with Wnt3A. **g** qPCR analyses of Tbx5, Hcn4, Tbx1, and Fgf10 after induction with Bmp4 (1.25 ng/mL) alone or in combination with Wnt3a (100 ng/mL), Wnt5A (100 ng/mL), Wnt11 (100 ng/mL), and IWP-2 (0.5 μM). **h** Representative FACS plots of Hcn4-GFP^+^ and Tbx1-cre, RFP^+^ CPCs at day 5.5. **i** qPCR analyses of Tbx5, Hcn4, Tbx1, and Fgf10 after induction with Bmp4 (1.25 ng/mL) alone or in combination with Noggin (100 ng/mL) or dorsomorphin (100 nM), K2288 (100 nM), and DMH1 (100 nM). **j** Topflash assay after induction (88 h of differentiation) with Bmp4 (1.25 ng/mL) or Wnt3A (100 ng/mL) alone or in combination with Wnt3a, IWP-2, Noggin or Dorsomorphin. All data are mean ± SEM; *n* = 3; *p < 0.05; **p < 0.01. *p* values were determined using a paired Student’s *t* test

To gain mechanistic insights into inductive signals of heart fields, we performed ingenuity pathway analysis (IPA)^41,42^ on the lists of 592 and 1377 genes that were differentially upregulated in the GFP^+^ and RFP^+^ cells, respectively. IPA utilizes an input gene list and a curated database of literature-derived pathways to infer which canonical pathways are most significant to the input data set. We focused our analysis on pathways related to “organism growth and development”. IPA inferred that activity of Actin cytoskeleton, Paxillin, Notch and Bmp signaling pathways are enriched in GFP^+^ cells while Wnt activity was enriched in RFP^+^ cells (Fig. 3a, Supplementary Data 1). The high activity of Actin cytoskeleton and Paxillin signaling pathways likely reflects the presence of structural genes in FHF cells. Notably, the key members of Bmp or Wnt/β-catenin signaling components—BmpR1a, Bmp2 and Bmp4 or Axin2, Fzd, and Dkk1—were upregulated in GFP^+^ or RFP^+^ cells, respectively (Fig. 3b).

We initially evaluated the effect of Bmp signals during heart field specification. In order to minimize the possibility of influencing heart field cells after induction, all of the data were analyzed within 12 h after the appearance of GFP^+^/RFP^+^ cells. We found that increasing Bmp4 levels promote induction of both GFP^+^ cells and RFP^+^ cells, but only GFP^+^ cells responded in a dose-dependent manner (Fig. 3c, d). RFP^+^ cells were also induced, but their induction was generally maintained except at the highest concentration (Fig. 3d). On the other hand, increasing levels of Activin A, a key ligand for Activin/TGF-β signaling, had no apparent effect (Supplementary Fig. 4a). This suggests that Bmp signaling promotes FHF specification and may allow SHF specification. Interestingly, increasing concentrations of Wnt3A correlated with increased numbers of RFP^+^ cells but did not affect GFP^+^ cells (Fig. 3e). This indicates that Wnt signaling specifically promotes specification of the SHF. Intriguingly, the observed Bmp4-mediated induction of RFP^+^ cells was abolished by the porcupine inhibitor IWP-2, a potent inhibitor of Wnt secretion (Fig. 3f). This suggests that Bmp signaling specifies the SHF via endogenous Wnt ligands.

To investigate the crosstalk between Bmp and Wnt signals, we treated the spheroids with Bmp4 and Wnts in combinations and analyzed expression levels of FHF (*tbx5, hcn4*) and SHF (*tbx1, fgf10*) markers. Similar to the earlier finding, Bmp4 alone increased expression of both heart field markers, but SHF marker expression was suppressed, when IWP-2 was added (Fig. 3g). Likewise, the addition of Wnt3A resulted in a further increase of the SHF markers and a reduction of the FHF markers (Fig. 3g). The combination with Wnt5A or Wnt11 caused an overall reduction of all markers (Fig. 3g), indicating that noncanonical Wnts signaling do not regulate heart field specification. These data suggest that Bmp signaling may increase canonical Wnt signaling for SHF specification. To test this, we measured canonical Wnt activity with its readout Topflash. Indeed, treatment with Bmp4 alone increased topflash activity, and the activity was further increased when the cells were treated in combination of Bmp4 and Wnt3A (Fig. 3j).

Based on our finding that Bmp signals promote both heart field specification and Wnt activity, we further tested if Bmp signals are necessary for these events, done by treating the spheroids with Noggin, which blocks Bmps from binding their receptors^43^. The treatment abolished Bmp’s inductive effects on GFP^+^/RFP^+^ cells, accompanied with markedly reduced Wnt activity (Fig. 3h). Since Bmp-mediated induction of RFP^+^ cells requires Wnt signaling, these data suggest that Bmp signaling is required and sufficient for specifying both FHF and SHF cells and activates Wnt signaling for the SHF specification. Notably, dorsomorphin, DMH1, and K2288, selective Bmp type I receptor inhibitors of SMAD-dependent signaling^44,45^, suppressed GFP^+^ cell induction and FHF genes without significantly affecting RFP^+^ cells, SHF genes, and Wnt activity (Fig. 3h–j). This was further supported by the co-treatment of cardiac spheroids with Noggin or dorsomorphin, which showed inhibition or no effect, respectively, on the Bmp-mediated increase in topflash activity. Together, these data suggest that FHF cells are specified through the BMP/SMAD pathway, whereas SHF cells are specified via a SMAD-independent BMP/Wnt pathway.

### Cxcr4 identifies SHF progenitors in vivo and in vitro

**Fig. 4:**
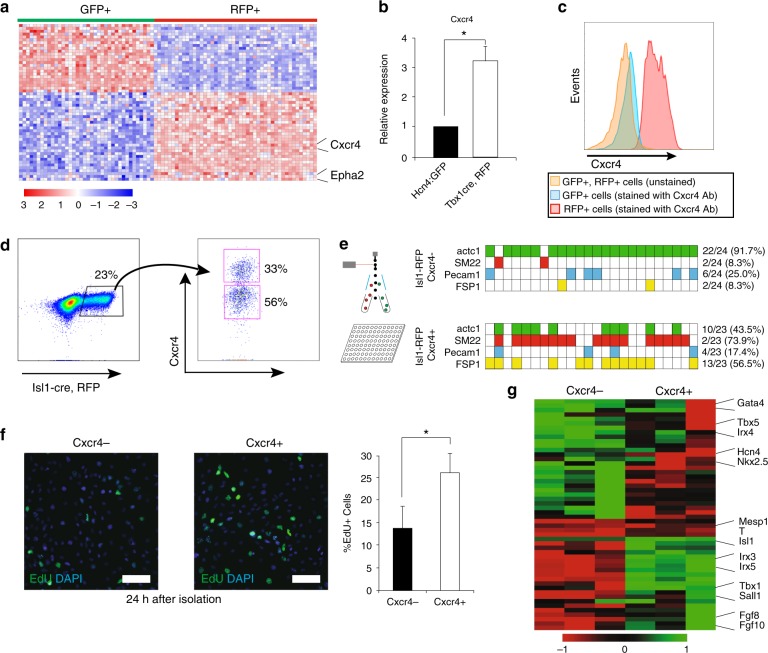
Cxcr4 marks SHF progenitors in mouse PSC-derived spheroids and in embryos. **a** RNA-seq analysis of differentially expressed surface receptors between Hcn4-GFP^+^ and Tbx1-cre, RFP^+^ CPCs in vitro. We identified 240 differentially expressed surface receptors of which the top 55 are shown here (all with Benjamini–Hochberg adjusted *p*-value < 0.05). **b** qPCR analysis of Cxcr4 expression in Hcn4-GFP + and Tbx1-cre, RFP + CPCs. Data are mean ± SEM; *n* = 3; *p < 0.05; *p* values were determined using a paired Student’s *t* test. **c** Representative flow histograms of Cxcr4 staining of Hcn4-GFP^+^ and Tbx1-cre, RFP^+^ CPCs compared to unstained GFP^+^/RFP^+^ CPCs. **d** Representative flow cytometric analysis and sorting strategy of Isl1-cre, RFP^+^ CPCs (left) and Cxcr4^−/+^ CPCs (right). **e** Clonal cell-fate assay showing qPCR results from 2 × 24 single-cell Cxcr4^−^ and Cxcr4^+^ clones, sorted and plated at day 5.5 and analyzed 7 days later. Solid colors represent expression of the gene of interest (Ct value < 30). Green: cTnT, Red: SM22, Blue: Pecam1, Yellow: FSP1/S100A4. **f** Cxcr4^−^/+CPCs isolated day 5.5, treated with EdU 24 h after isolation and stained for EdU and DAPI. Scale bars represent 50 μm, bar graph shows quantification of EdU + CPCs, Data are mean ± SEM; *n* = 4; *p < 0.05; *p* values were determined using a paired Student’s *t* test. **g** Microarray analysis showing expression of cardiac genes in Isl1-cre, RFP^+^ vs. Isl1-cre, RFP^-^ CPCs isolated day 5.5

Developing a non-genetic way to identify and isolate specific cell types is crucial for PSC-based regenerative medicine. We therefore searched for cell surface markers enriched in FHF or SHF cells. By RNA-sequencing analysis, we identified 240 differentially expressed surface receptors between GFP^+^ and RFP^+^ cells (Fig. 4a). Given that SHF cells are migratory^37,46^, we focused on genes involved in cell mobilization and identified the two receptors C-X-C Chemokine Receptor type 4 (Cxcr4) and Ephrin type-A receptor 2 (EphA2), which were both upregulated in the RFP^+^ cells compared to GFP^+^ cells in vitro. Their differential expression was confirmed by qPCR in vivo and in vitro (Fig. 4b, Supplementary Fig. 5a). In order to determine the expression in vivo, we analyzed expression of Cxcr4 and Epha2 along with other cardiac markers in the Mesp1-derived progeny in the mesodermal core of the 2nd pharyngeal arch at E9.0, which harbors undifferentiated and expansive SHF-CPCs^37^. To do this, we dissociated arches from *Mesp1*^*Cre*^; *Ai9* mice and isolated RFP^+^ and RFP^−^ cells by FACS followed by qPCR analysis. Both Cxcr4 and Epha2 were significantly enriched in RFP^+^ CPCs compared to RFP^-^ cells (Supplementary Fig. 5a). While *Epha2* levels were increased in the developing heart, the *Cxcr4* expression pattern was similar to that of undifferentiated CPC markers (*Tbx1, Fgf10, Isl1*), indicating that Cxcr4 exclusively marks undifferentiated SHF-CPCs (Supplementary Fig. 5a). We further confirmed the co-expression of Isl1 and Cxcr4 in the mesodermal core of PA2 by immunohistochemistry (Supplementary Fig. 5b). Additionally, FACS analyses of GFP^+^/RFP^+^ CPCs at day 5.5 confirmed that Cxcr4 exclusively marks Tbx1-Cre, RFP^+^ but not Hcn4^−^GFP^+^ CPCs in cardiac spheroids (Fig. 4c).

To determine whether Cxcr4 marks SHF-CPCs in vitro, we generated an ESC line from *Isl1*^*Cre*^; *Ai9*; *MHC*^*GFP*^ mice in which RFP permanently marks Isl1 progeny and cardiomyocytes can be identified by GFP expression^47^. After 5.5 days of differentiation, Cxcr4 identified a subset of RFP^+^ CPCs (Fig. 4d, Supplementary Fig. 5d). RFP^+^/Cxcr4^−^ or Cxcr4^+^ cells were FACS- isolated with Cxcr4 antibody and analyzed with qPCR. Accordingly, the FHF markers *Hcn4, Tbx5, Nkx2.5*, and *Gata4* were enriched in Isl1-Cre, RFP^+^/Cxcr4^−^ CPCs, whereas the SHF markers *Fgf10* and *Tbx1* were enriched in RFP^+^/Cxcr4^+^ cells (Supplementary Fig. 5c). *Isl1* levels were not significantly different between Cxcr4^+^ and Cxcr4^−^ CPCs, similar to the expression levels in Hcn4^−^GFP^+^, Tbx1-Cre, RFP^+^ CPCs (Supplementary Fig. 5c). In order to determine the cardiac differentiation potential of the two populations, single RFP^+^/Cxcr4^−^ or Cxcr4^+^ cells were isolated from day 5.5 spheroids and clonally expanded for 7 days. We found that RFP^+^/Cxcr4^−^ cells primarily differentiated into cardiomyocytes, while RFP^+^/Cxcr4^+^ cells gave rise to multiple cardiac lineages (Fig. 4e). RFP^+^/Cxcr4^+^ cells were more proliferative than RFP^+^/Cxcr4^−^ cells, determined by nucleoside 5-ethynyl-2′-deoxyuridine (EdU) incorporation (27% vs. 14%) (Fig. 4f). The cellular identities of Cxcr4^+^ or Cxcr4^−^ cells were further confirmed by microarray analysis (Fig. 4g, Supplementary Data 3). These data suggest that PSC-derived FHF or SHF cells can be distinguished and purified based on their expression of Cxcr4. By qPCR we confirmed that *Epha2* levels were elevated in Cxcr4^+^ CPCs. Likewise, FACS analyses demonstrated that Epha2 marked a subset of RFP^+^ CPCs similar to Cxcr4. Accordingly, Cxcr4 levels were increased in RFP^+^, Epha2^+^ cells while Tbx1 and Tbx5 showed a similar expression pattern to that of RFP^+^, Cxcr4^+^ CPCs, implying that Cxcr4 and Epha2 marks the same population of SHF CPCs. Finally, we validated the cardiac disease modeling potential in Isl1-Cre, RFP^+^, Cxcr4^+/−^ CPC populations by knocking down *Tbx5* and *Tbx1*. Importantly, Tbx5 knockdown only affected cardiogenesis in Cxcr4^−^ CPCs (Supplementary Fig. 5e), whereas Tbx1 knockdown only had an effect on Cxcr4^+^ CPCs (Supplementary Fig. 5f), similar to the knockdown experiments in Hcn4^−^GFP^+^ and Tbx1-Cre, RFP^+^ CPCs (Supplementary Fig. 2h, i).

Taken together, these results demonstrate how Cxcr4 and Epha2 expression identifies undifferentiated SHF-CPCs, and how Cxcr4 and EphA2 may be used to develop non-genetic approaches to isolate undifferentiated CPCs from mouse PSC cultures.

### CXCR4 identifies SHF progenitors in human iPSC spheroids

**Fig. 5:**
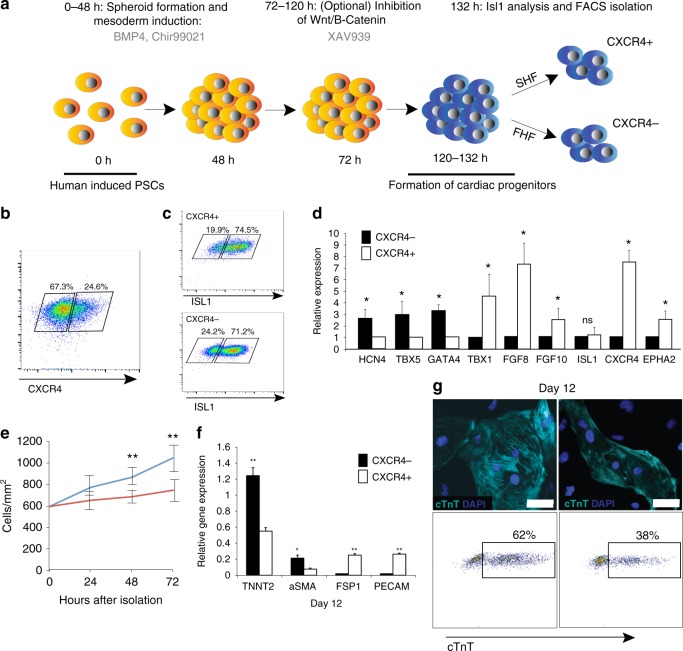
CXCR4 identifies SHF progenitors in human iPSC-derived spheroids. **a** Schematic of the strategy used to generate and differentiate hiPSC-derived cardiac spheroids. **b** Representative flow cytometric analyses showing the number of CXCR4^−^ and Cxcr4^+^ cells at day 5.5. **c** Representative flow cytometric analysis showing the number of human Isl1^+^ cells in the sorted populations of CXCR4^+^ and CXCR4^−^ CPCs. **d** qPCR analyses of CXCR4^−^ and CXCR4^+^ cells isolated at day 5.5. Data are mean ± SEM; *p < 0.05; **p < 0.01; ns not significant (*p* > 0.05). *p* values were determined using a paired Student’s *t* test. **e** Cell counts of CXCR4^−^ and CXCR4^+^ cells isolated day 5.5. Data are mean ± SEM; **p < 0.01. *p* values were determined using a paired Student’s *t* test. **f** qPCR analysis of Tnnt2, aSMA (smooth muscle cell marker), Fsp1 (fibroblast marker), and PECAM (endothelial cell marker) in cells derived from Cxcr4^−^ and CXCR4^+^ CPCs isolated at day 5.5, re-plated as monolayers and isolated at day 12. Data are mean ± SEM; *n* = 3 *p < 0.05; **p < 0.01; ns not significant (*p* > 0.05). *p* values were determined using a paired Student’s *t* test. **g** Representative images of human cardiomyocytes from CXCR4^−^ and CXCR4^+^ derived cardiomyocytes at day 12 (Above) and representative flow cytometric analyses of cTnT^+^ cardiomyocytes at day 12 (Below). White scale bars indicate 25 μm

To determine whether two heart fields are induced in human PSCs, we devised a protocol for hiPSCs to generate spheroids based on Bmp4 and Wnt activation with the small-molecule inhibitor Chir99021 that allows induction of high percentages of ISL1 CPCs^48^ (Fig. 5a). At day 5.5, we isolated CXCR4^−^ and CXCR4^+^ cells from the spheroids by FACS and approximately 75–80% of cells in both populations expressed the CPC marker ISL1, indicating a commitment to the cardiac lineage (Fig. 5b, c). In addition, we analyzed the expression of heart field genes in the sorted CPCs. Similar to the mouse PSC system, the FHF genes (*HCN4*, *TBX5, GATA4*) or the SHF genes (*TBX1*, *FGF10, FGF8*) were highly upregulated in CXCR4^−^ cells or CXCR4^+^ cells, respectively (Fig. 5d, Supplementary Data 2). The CXCR4^+^ cells were more proliferative than CXCR4^−^ cells while CXCR4^−^ cells were more cardiomyogenic than CXCR4^+^ cells (62% vs. 38%) (Fig. 5e–g). After differentiation, CXCR4^+^ cell progeny expressed high levels of smooth muscle, endothelial, and fibroblast markers (Fig. 5f and Supplementary Fig. 6), supporting their multipotency, while cells derived from CXCR4^−^ CPCs expressed high levels of cardiomyocyte markers and low levels of endothelial/fibroblast markers (Fig. 5f). These data suggest that FHF and SHF cells are generated and distinguished by CXCR4 expression in human iPSC spheroids.

## Discussion

In the current study, we used mouse and human PSCs to model the earliest stages of heart field development, with the goal to identify the inductive signals of the two heart fields and to create a model system that allows the study of heart field-specific developmental events. The derivation of embryonic stem cells from developing *Hcn4-GFP; Tbx1-Cre; Ai9* embryos allowed us to directly compare CPCs between in vivo and in vitro, and thereby use mouse embryos as reference for heart field specification in vitro. In particular, the use of the *Tbx1-Cre* allele allowed us to trace and follow RFP^+^ progeny in the spheroid system. While an earlier study used a two-reporter system with Mef2c/Nkx2.5 enhancer-driven RFP/GFP, the analysis was done at a later stage (E9.5), when the heart is present^49^. The findings from this work provide a scheme of which distinct heart field populations are specified during gastrulation by gradients of Bmp and Wnt/β-catenin signaling and can be identified by based on Cxcr4 expression (Supplementary Fig. 7). We propose that the FHF is induced by Bmp/Smad signaling during gastrulation stage, whereas the SHF is induced by Bmp-mediated activation of canonical Wnt signaling. Collectively, these new insights are expected to provide a framework for studying the earliest stages of mammalian cardiac development and a platform for efficient generation of chamber-specific progenitors for human iPSC-based heart disease modeling.

Our findings that the LIM homeodomain transcription factor Isl1 progeny give rise to the entire heart is supported by several earlier studies^31,34,35,50^. Isl1 has been regarded a SHF marker since it was first described in a fate-mapping study with *Isl1*-*IRES-Cre* mice, where Cre was inserted into the exon encoding a LIM domain^21,36^, and since Isl1-null embryos primarily affect development the OFT and RV^21^, indicating that Isl1 plays an essential role in development of the SHF and structures derived thereof. However, retrospective lineage tracing experiments using an efficient *Isl1*-Cre knock-in mouse line showed that most cells in the LV also originate from Isl1-expressing cells^31^. Other studies have reported that Isl1 protein is expressed at E7.5 throughout the anterior intra-embryonic coelomic walls and proximal head mesenchyme, regions that encompass both the FHF and the SHF in mouse^34^, and more recently, that Isl1 is expressed in Tbx5-expressing cells isolated from the cardiac crescent^35^, implying that Isl1 may be temporarily expressed in both heart fields. It has been suggested that the inefficient recombination activity of the original *Isl1*-IRES-Cre might have contributed to the conclusion made earlier^51^. We therefore conclude that Isl1 is a pan-cardiac marker, expressed in all undifferentiated CPCs similar to the transcription factor Sall1^29^.

Based on our observations and the FACS analyses, GFP^+^ and RFP^+^ cells appear invariably around the same time. We have not observed any specific cases when one reporter appears first. However, there are several developmental and technical considerations that make it difficult to conclude the order of their induction: first, while both of the FHF and the SHF appear at the cardiac crescent stage (E7.25–7.75), our and published studies suggest that their precursors might be specified during gastrulation (E6.5–7.0)^7,8^. Second, there might be a slight delay as RFP expression is activated upon Tbx1-Cre expression. Finally, Hcn4-GFP is a fusion protein emitting signals lower than RFP.

The concept that both heart fields are specified in nascent mesoderm is supported by two studies^7,8^, where FHF and SHF progenitors were shown to be present in two temporal pools of Mesp1-expressing cells during gastrulation. Although the fluorescent reporters we used to visualize the two heart fields are not activated during germ layer formation, the findings from our precardiac spheroid system clearly demonstrate that their specification is positively regulated by Bmp and Wnt signals during a gastrulation stage, which is defined by a temporal expression of Brachyury and Mesp1 (Supplementary Fig. 4b)^5,7,8,11^. It will be of great importance to determine the specific inductive roles of the two morphogens in heart field formation in vivo.

Curiously, increasing levels of Activin A did not have a significant effect on cardiogenesis and no overall effect on heart field induction. This may suggest a broader role of Activin A in mesoderm formation. This is supported by the previous report that signaling from the Activin A receptor Acvr1b regulates the fates of mesendoderm progenitors^13^. In fact, Acvr1b signaling was shown to favor endoderm formation by repressing expression of members of the Id family of DNA-binding protein inhibitors, whereas its reduction depresses Id genes and promotes cardiac mesoderm formation^13^. Bmp signaling directly activates transcription of Id1^52,53^, which is necessary and sufficient to induce cardiac differentiation in mouse and human PSCs via upregulation of FHF genes, but not SHF genes^13^. In addition, mice deficient of Id1-4 fail to express the FHF genes Smarcd3, Tbx5, and Nkx2.5 in the anterior region of the cardiac crescent^13^, suggesting that Bmp signaling may activate the FHF program through Id genes. Consistently, our RNA-sequencing analysis revealed that Id1, 2, and 4 were upregulated in Hcn4-GFP^+^ CPCs at day 5.5.

Activin A and Bmp4 were shown to play a pivotal role in generating distinct subpopulations of mesoderm in a human PSC system^54^. They are distinguished by expression of RALDH2 and CD235a/CYP26A1 and give rise to atrial and ventricular cardiomyocytes, respectively^54^. The specification of ventricular progenitors was dependent on a higher ratio of Activin A to Bmp4 signaling than one required for the atrial lineage^54^. We found that ALDH1A2 (RALDH2) was highly expressed in SHF CPCs both in vivo and in vitro. This may suggest that SHF progenitors contain RALDH2^+^ atrial progenitors.

The finding that Bmp4-mediated upregulation of canonical Wnt signaling is necessary for specification of multipotent cardiac progenitors provides new insights into how the distinct heart fields are specified. In vivo, Bmp4 and Wnt/β-catenin signaling plays critical roles in early cardiogenesis^55,56^. However, it remains unclear which cell types secrete Bmp and Wnt ligands and how these signals influence early heart field development. Our findings suggest that the Bmp4-receiving cells, giving rise to the FHF, may play an inductive role for SHF specification via positive regulation of expression of Wnt ligands. While evidence presented here show the presence of distinct pathways regulating these events, additional studies will be necessary to elucidate the precise mechanisms.

The ability to recapitulate and monitor heart field development in a PSC system has enabled us to investigate the molecular pathways that regulate early cardiac fate decisions. Our findings emphasize the importance of the PSC system in understanding the earliest stages of cardiac development. In fact, the system offers many advantages, such as an unlimited source to generate mesodermal cells, cell differentiation in a defined condition, and time-lapse capability, and can avoid the experimental difficulties associated with gastrulation-stage embryos such as size, staging, and quantity. While expression trend patterns between FHF and SHF corresponded very well between in vitro and in vivo, absolute expression values (for example, normalized counts) did not correspond well between in vivo and in vitro. This phenomenon is not unique to our study but rather observed frequently in in vitro, stem cell-derived tissue models^57^. It will be important to investigate how the values are differentially regulated in vitro.

There are several heart field/chamber-specific CHDs including hypoplastic left heart syndrome and hypoplastic right heart syndrome^27,58^ as well as some chamber-specific cardiomyopathies and tachyarrhythmias like arrhythmogenic right ventricular cardiomyopathy or right ventricular outflow track ventricular tachycardia^59,60^. The pathogenesis of these diseases has remained unexplored to a significant extent, partly due to the inability to obtain cardiac tissue from patients. Thus, our method is expected to offer a unique opportunity to study heart field/chamber-specific cardiac diseases using patient derived transgene-free CPCs.

## Methods

### Generation of Hcn4-GFP, Tbx1-Cre, Ai9 mice, and ESCs

Hcn4-GFP, Tbx1C, Ai9 mice were obtained by crossing Hcn4-GFP mice^32^ with Tbx1-Cre mice^17^ and Ai9 reporter mice (stock no. 007909, Jackson Laboratory). The appearance of the vaginal plug was considered as day 0.5 of gestation (E0.5). Mouse ESCs^*Hcn4-GFP; Tbx1-Cre; Ai9*^ were derived from blastocysts (E3.5) harboring Hcn4-GFP; Tbx1-Cre; Ai9 and mESC^*Isl1-Cre, α MHC-GFP -GFP; Ai9*^ were derived from blastocysts (E3.5) harboring Isl1-Cre^36^, αMHC-GFP^47^; Ai9. All animals were housed at the Johns Hopkins Medical Institutions. All protocols involving animals followed U.S. NIH guidelines and were approved by the animal and care use committee of the Johns Hopkins Medical Institutions.

### Cell work

Mouse ESCs and human iPSCs were maintained and differentiated as previously described^5,37,61^. Briefly, mESCs were maintained on gelatin-coated dishes in maintenance medium (Glasgow minimum essential medium supplemented with 10% fetal bovine serum and 3 μM Chir99021 and 1 μM PD98059 or 1000 U/mL ESGRO (Millipore, Billerica, MA), Glutamax, sodium pyruvate, MEM non-essential amino acids (Thermo Fisher Scientific). For spheroid formation and differentiation, mouse ESCs were plated in IMDM/Ham’s F12 (Cellgro) (3:1) supplemented with N2, B27, penicillin/streptomycin, 2 mM GlutaMAX, 0.05% BSA, 5 ng/mL l-ascorbic acid (Sigma-Aldrich) and α-monothioglycerol (MTG; Sigma-Aldrich) at a final density of 100,000 cells/mL to allow spheroid formation. After 48 h spheroids were collected and transferred to ultra-low attachment plastic surface and induced for 40 h with Activin A, Bmp4, Wnt3A, Wnt5A, Wnt11 (R&D Systems) alone or in combination. Human iPSCs were maintained in Geltrex-coated T25 flasks using Essential 8 medium. For spheroid formation and differentiation, hiPSCs were plated in RPMI plus B27 minus insulin with Bmp4 and CHIR99021 and incubated for 48 h. After 48 h media was changed to RPMI plus B27 minus insulin for 24 h followed by 48 h of treatment with XAV939 (Sigma-Aldrich). The hiPSC line used in this study was previously developed by Dr. Shinya Yamanaka’s laboratory^62^.

### siRNA, transfection, and luciferase assays

For *Tbx1 and Tbx5* knockdown experiments, Tbx1 and Tbx5 ON-TARGETplus SMARTpool siRNA or scrambled siRNA (Dharmacon/Thermo Fisher Scientific) was used at 5 nM for cell transfection. Cells were transfected with Lipofectamine LTX (Life Technologies) in single-cell suspensions. For TOP-flash luciferase assays, mESCs were transfected with Topflash constructs and renilla constructs and analyzed as previously described^5^.

### Live cell imaging, EdU labeling, immunohistochemistry, and microscopy

For live imaging, single cardiac organoids were plated in round bottom ultra-low plates (Cat# 7007, Corning, Inc). Each well was imaged every hour for GFP and RFP expression up to 96 h using a BZ-9000 Fluorescence Microscope (Keyence). For EdU analysis, Click-it EdU kit (Life Technologies) was used followed by immunostaining with primary and secondary antibodies. For whole-mount staining, embryos were fixed in 4% paraformaldehyde overnight and then 30% sucrose and then incubated with primary and secondary antibodies. For immunohistochemistry, embryos were fixed in 4% paraformaldehyde overnight and then 30% sucrose, and then embedded in OCT, sectioned and stained using standard protocols. Antibodies used were: mouse α-Islet1 (1:200; Cat. 39.3F7 Developmental Studies Hybridoma Bank, Iowa City, IA), rat α-RFP (1:200; Cat. 5F8 Chromotek), chicken GFP (1:500; Cat A10262 Invitrogen), rabbit Cxcr4 (1:500; Cat. 119-15995 Biotrend), rabbit αSMA (1:200; Cat. Ab5694 Abcam), Pecam-1 (1:100; Cat. 553371 BD Biosciences), Thy1 (Cat. 17-0902-82 eBiosciences), mouse cardiac TnT (1:500; Cat. MS-295-P1Thermo Fisher). Alexa Fluor secondary antibodies (1:500; Life Technologies) were used for secondary detection and images were acquired with an Evos fl microscope.

### Flow cytometry and cell sorting

Mouse embryos (E7.75) were dissected using forceps under a stereomicroscope (Zeiss) and regions of interest were dissociated and harvested using TrypLE. Embryoid bodies (EBs) and cells were dissociated and harvested using TrypLE. Single-cells were analyzed for RFP/GFP expression or sorted using a SH800 Cell sorter (Sony Biotechnologies). Live cells were analyzed for RFP and GFP expression and stained with antibodies targeting for the presence of appropriate markers. Cells were stained with the following antibodies: anti-mouse Cxcr4 conjugated with PerCP-eFluor 710 (1:200; 46-9991-80 eBiosciences) anti-mouse EphA2 conjugated with APC (1:100; Cat. FAB639A R&D systems), anti-human Cxcr4 conjugated with PE or APC (1:25; Cat. FAB170P R&D systems). For cTNT and Isl1 expression, cells were fixed with 4% paraformaldehyde (PFA) for 10 min, permeabilized with saponin (Sigma), stained with either mouse cTNT (1:500, Cat. MS-295-P1 Thermo Scientific) or mouse Islet1 antibody (1:200, Cat. 39.3F7 Developmental Studies Hybridoma Bank, Iowa City, IA), followed by incubation with secondary antibody conjugated with Alexa Fluor 647 (1:500, Invitrogen). Data were analyzed using FlowJo software.

### Quantitative RT-PCR

RNA isolation was performed using either RNeasy Micro Kit (Cat# 74004, Qiagen) or ARCTURUS® PicoPure® RNA Isolation Kit following the manufacturer’s instructions, and cDNA was generated using the high-capacity cDNA reverse transcription kit (Applied Biosystems). qPCR reactions were performed using the Taqman (Applied Biosystems) or Sybr Select qPCR mix (Thermo Fisher) with indicated primers. Gene expression levels were normalized to *Gapdh*. For the clonal cell-fate analysis, single Isl1-Cre RFP^+^, Cxcr4^−^, and Isl1-Cre RFP^+^, Cxcr4^+^ cells were sorted at day 5.5 into 384-well plates and allowed to grow and differentiate for 7 days. Appearance of colonies was visually confirmed by microscopy. RNA was isolated from 24 wells with colonies from Cxcr4^−^ and Cxcr4^+^ sorted cells, respectively. Ct values < 30 were considered positive. All samples were also analyzed for gapdh to exclude false-positive results.

### Library preparation and sequencing

GFP^+^ and RFP^+^ cells were isolated using a SH800 cell sorter (Sony Biotechnologies) into 96 plates containing water (2.4 mL) with RNase-free DNase I (0.2 mL; NEB) and RNase inhibitor (0.25 mL; NEB). Each sample represents 10 cells. DNase I was inactivated by increasing the temperature (72 °C for 3 min), and samples were then stored on ice. Custom-designed 2 A oligo 1-mL primer (12 mM, Integrated DNA Technologies^25,63^ was added and annealed to the polyadenylated RNA by undergoing a temperature increase (72 °C for 2 min) and being quenched on ice. A mixture of 1 mL of SMARTscribe reverse transcriptase (Clontech Laboratories), 1 mL of custom-designed TS oligo (12 mM, Integrated DNA Technologies^63^, 0.3 mL of MgCl_2_ (200 mM, Sigma), 0.5 mL of RNase inhibitor (Neb), 1 mL of dNTP (10 mM each, Thermo), and 0.25 mL DTT (100 mM, Invitrogen) were incubated at 42 °C for 90 min, which was followed by enzyme inactivation at 70 °C for 10 min. A mixture of 29 mL of water, 5 mL of Advantage2 taq polymerase buffer, 2 mL of dNTP (10 mM each, Thermo), 2 mL of custom-designed PCR primer (12 mM, Integrated DNA Technologies^63^, and 2 mL of Advantage2 taq polymerase was directly added to the reverse transcription product, and the amplification was performed for 19 cycles. The amplification product was purified using Ampure XP beads (Beckman-Coulter). Libraries and transposome assembly were made using a previously published protocol^64^. Briefly, 100 pg of total cDNA was added to a 2× tagment DNA Buffer (TD) (2× TAPS buffer: 20 mM TAPS-NaOH, 10 mM MgCl_2_ (pH 8.5) at 25 C, and 16% weight volume (w/v) PEG 8000), and then spiked with 0.5 mL of 1:64 diluted Tn5 (Epicenter) and incubated for 8 min at 55 C. Tn5 was stripped off from the tagmented DNA by adding 0.2% SDS for a final concentration of 0.05%. Libraries were enriched used KAPAHiFi, which included 5X Kappa Fidelity Buffer, 10 mM dNTPs, and HIFI polymerase, and 1 μL of index primers was used directly in the enrichment PCR amplification of libraries for the Illumina sequencers for a 50-mL reaction. The PCR program was as follows: 5 min at 72 °C and 1 min at 95 °C, and then 16 cycles at 30 s at 95 °C, 30 s at 55 °C, 30 s at 72 °C, and 5 min at 72 °C. For analysis, raw sequencing reads were trimmed using Trimmomatic (0.36) with a minimum quality threshold of 35 and minimum length of 36 ^65^. Processed reads were mapped to the mm10 reference genome using HISAT2 (2.0.4)^66^. Counts were then assembled using Subread featureCounts (1.5.2) in a custom bash script^67^. Differential gene expression analysis was done using the DESeq2 package in R^68^. Gene ontology analysis was performed using the PANTHER Version 12.0 classification^69,70^. Canonical pathway analysis was done using Ingenuity Pathway Analysis (QIAGEN Inc., https://www.qiagenbioinformatics.com/products/ingenuitypathway-analysis). To perform surface receptor analysis, list of candidate surface receptors was identified from the UniProtKB/Swiss-Prot database using the search terms “Gene Ontology: transmembrane signaling receptor activity” and “Organism: mus musculus.”

### RNA-sequencing analysis

Raw-sequencing reads were trimmed using Trimmomatic (0.36) with a minimum quality threshold of 35 and minimum length of 36 ^65^. Processed reads were mapped to the mm10 reference genome using HISAT2 (2.0.4)^66^. Counts were then assembled using Subread featureCounts (1.5.2) in a custom bash script^67^.

### Statistical analyses

All studies were done with at least three sets of independent experiments. Two-group analysis used Student’s *t* test. Comparisons of multiple groups were performed using either one-way or two-way ANOVA. *p* value < 0.05 was considered significant. For RNA-seq analysis, Benjamini–Hochberg correction was used to adjust for multiple testing, with threshold of adjusted *p*-value < 0.1 (i.e., false discovery rate < 10%) considered significant.

### Data availability

RNA-sequencing data that support the findings of this study have been deposited in GEO with the accession code “GSE116128”. The authors declare that all other data supporting the findings of this study available within the paper.

## Electronic supplementary material

Supplementary Information

Description of Additional Supplementary Files

Supplementary Movie 1

Supplementary Data 1

Supplementary Data 2

Supplementary Data 3
