# Supplementary material for: Precardiac organoids form two heart fields via Bmp/Wnt signaling
Source: Nat Commun. 2018 Aug 7;9:3140. doi: 10.1038/s41467-018-05604-8 (PMC6081372; doi:10.1038/s41467-018-05604-8)
Supplement: Supplementary file 1 — Supplementary Information [file 41467_2018_5604_MOESM1_ESM.pdf]

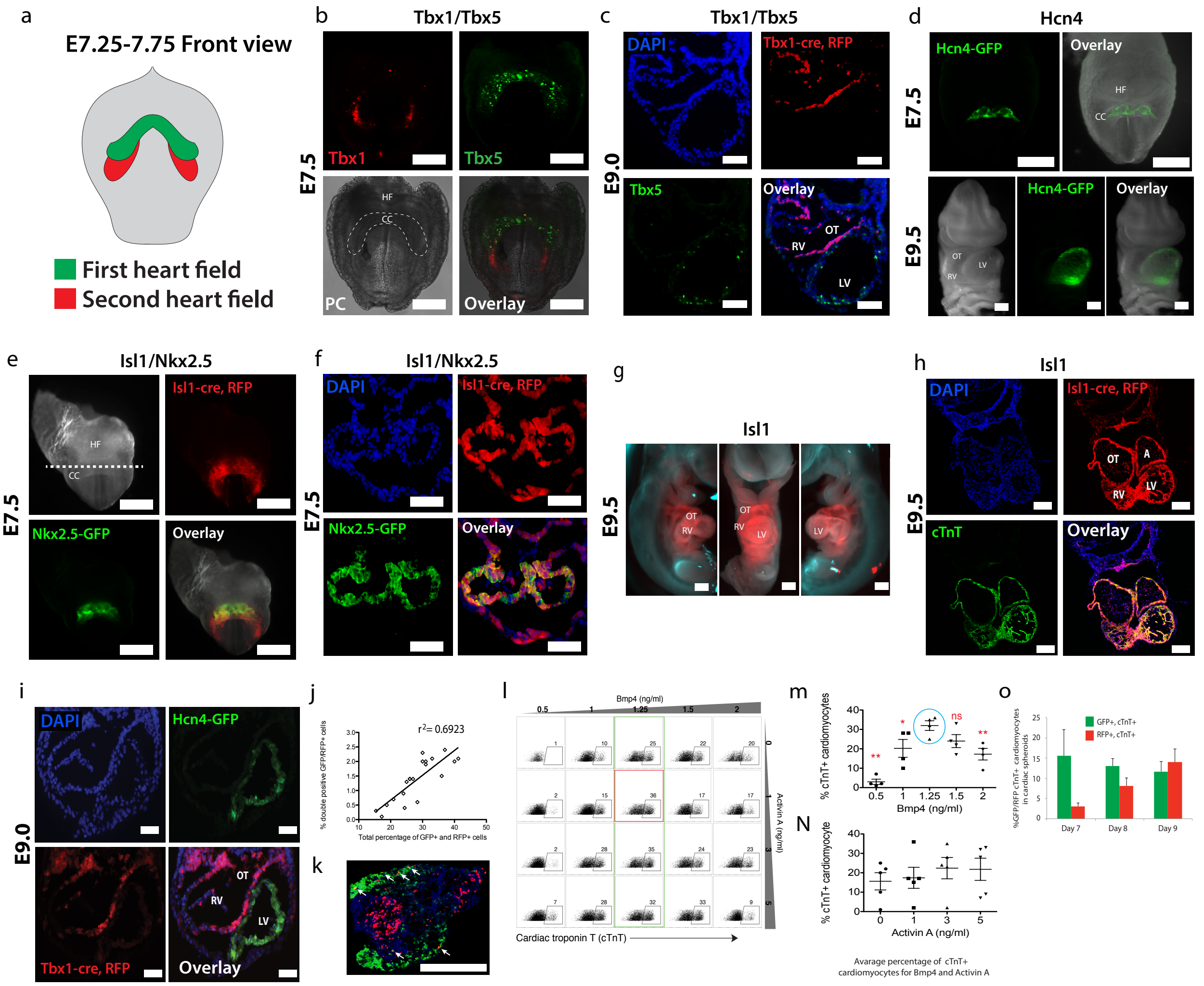

Supplementary Figure 1: Validation of FHF/SHF markers, ESC derivation, optimization of cardiac differentiation in vitro (a) Illustration of FHF and SHF localization at cardiac crescent stage (E7.25-7.75) (**b**) Tbx1 (red) and Tbx5 (green) wholemount staining at E7.5. (**c**) Tbx1-cre linage trace analysis (red) and Tbx5 (green) staining in transverse E9.0 section. (**d**) Hcn4-GFP at E7.5 (top) and E9.5 (bottom). (**e**) Wholemount Isl1-cre linage trace analysis (red) and Nkx2.5-GFP (green) at E7.5. (**f**) Isl1-cre linage trace analysis (red) and Nkx2.5-GFP (green) staining in transverse E9.0 section of embryo in (**e**). (**g**) Wholemount Isl1-cre linage trace analysis (red) E9.5. (**h**) Isl1-cre linage trace analysis (red) and cTnT (green) staining in transverse E9.0 section. (**i**) Tbx1-cre linage trace analysis (red) and Hcn4-GFP (green) staining in transverse E9.0 section from embryo in Fig. 1b. (**j**) Correlation between GFP^{+}/RFP^{+} double positive cells and total number of GRP^{+} and RFP^{+} cells (**k**) Section of a spheroid day 7. White arrows indicate double positive cells. (**l**) Flow cytometric analyses of cTnT in cardiac spheroids at day 9 of differentiation. (**m**) Vertical scatter plot of cTnT+ percentages in response to overall increasing Bmp4 concentrations. Data are mean ± SEM; *$p < 0.05$, **$p<0.01$, ns, not significant; p values were determined using one-way ANOVA analysis compared to 1.25ng/ml Bmp4. (**n**) Vertical scatter plot of cTnT+ percentages in response to overall increasing Activin A concentrations. (**o**) Percentage of GFP^{+}, $cTnT^{+}$ and RFP^{+}, $cTnT^{+}$ cardiomyocytes in cardiac spheroids. Data are mean ± SEM; No individual sample was different compared to each other ($p>0.05$). p values were determined using one-way ANOVA analysis. White scale bars indicate 100 µm.

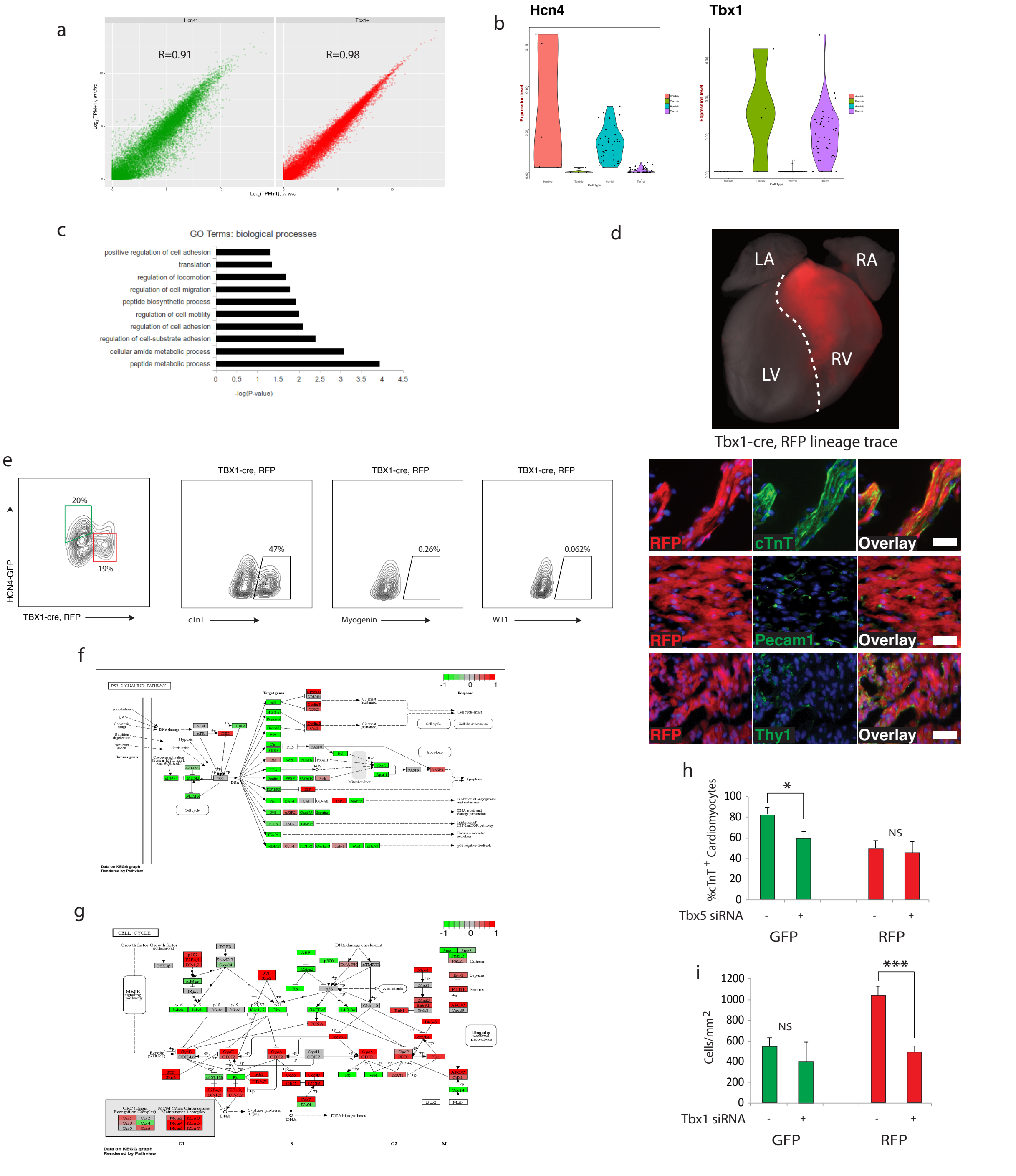

Supplementary Figure 2: RNA-sequencing analysis, Tbx1 lineage tracing, KEGG pathway analysis **(a)** Scatter plots showing in vitro vs. in vivo Hcn4-GFP+ cells (green) and Tbx1-Cre, RFP+ cells (red). (**b**) Hcn4 and Tbx1 violin expression level plots of in vitro and in vivo Hcn4-GFP$^+$ and Tbx1-Cre, RFP$^+$ samples. (**c**) GO terms analysis 585 genes that showed different expression patterns compared between in vitro and in vivo. (**d**) Tbx1-lineage trace of postnatal day 0 heart and immunohistochemistry analysis of cTnT, Pecam1 and Thy1 in Tbx1-Cre, RFP$^+$ structures. (**e**) Scatter plots showing GFP and RFP percentages at day 9 (left) and cTnT$^+$, Myogenin1$^+$ and WT1$^+$ cell percentages in RFP+ cells. (**f**) KEGG pathway analysis of cell cycle genes. White scale bars indicate 50 µm (**g**) KEGG pathway analysis of P53 signaling (red genes are upregulated in Tbx1-Cre, RFP$^+$ CPCs, green genes are upregulated in Hcn4-GFP$^+$ CPCs). (**h**) Percentages of cTnT$^+$ cardiomyocytes in GFP$^+$ and RFP$^+$ cells isolated and transfected with

siRNA against *tbx5* at day 5.5. Cells were analyzed 3 days after transfection. (**i**) Proliferation analysis of GFP^+ and RFP^+ cells 3 days after isolation and transfection with siRNA against *tbx1* at day 5.5. Data are mean ± SEM; $n = 3$; $*p < 0.05$, $***p < 0.001$. p values were determined using a paired Student's t test.

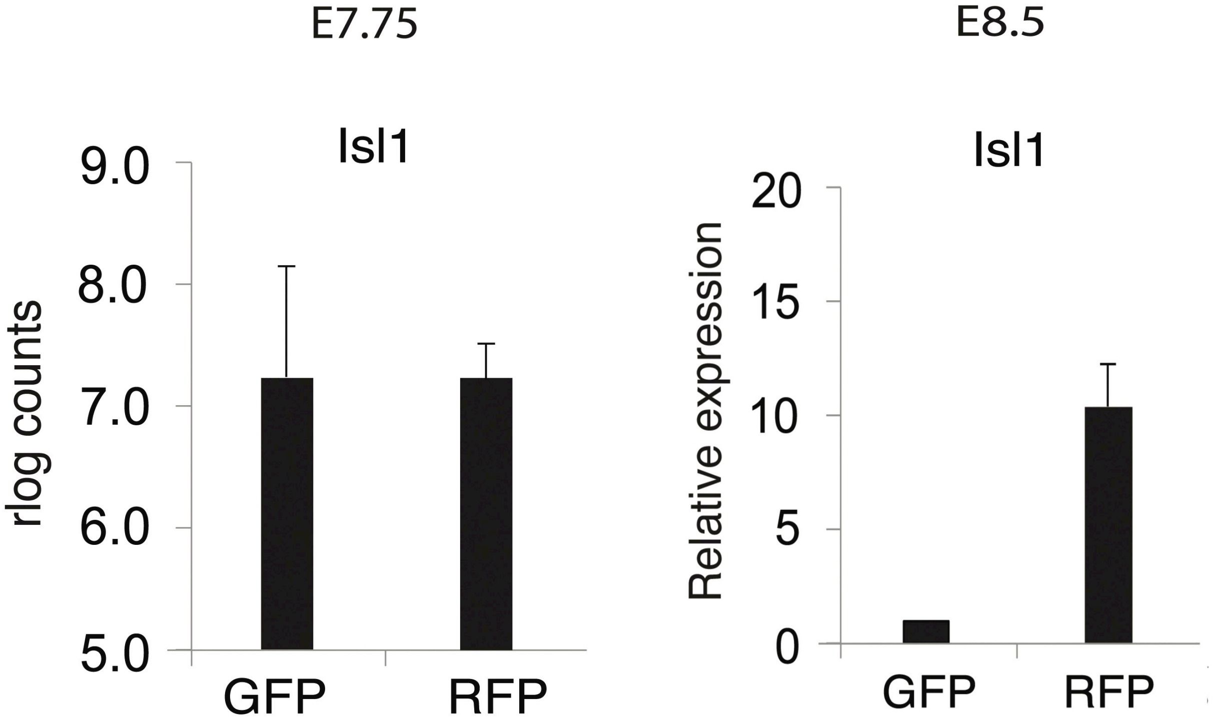

Supplementary Figure 3: ***Isl1*** **levels in GFP^{+} and RFP^{+} cells at E7.75 and E8.5.**

(**a**) Isl1 levels in GFP+ and RFP+ cells at E7.5. (**b**) Relative Isl1 levels at E8.5 in GFP+ and RFP+ cells.

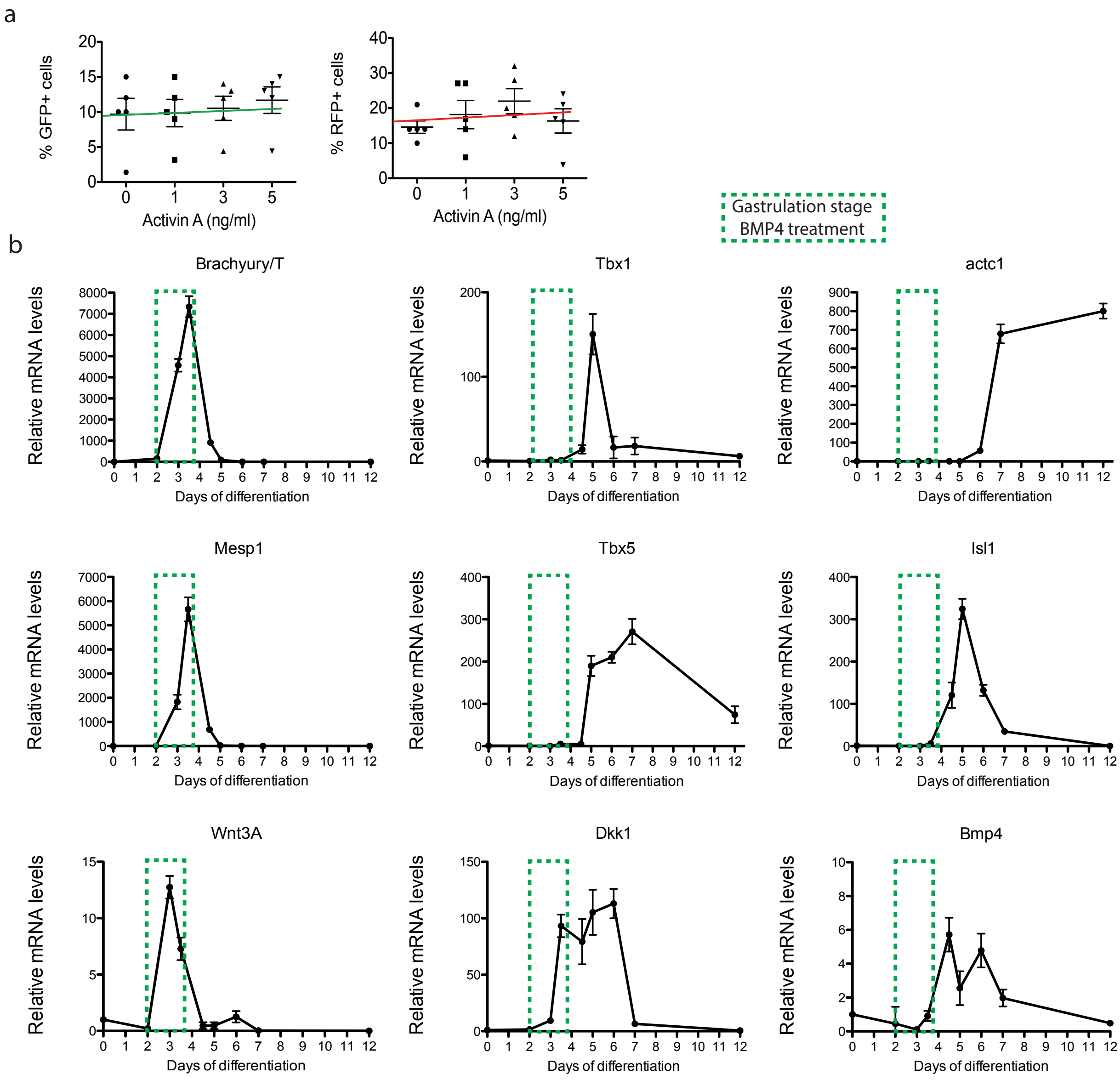

Supplementary Figure 4: Effect of Activin A on FHF/SHF specification and gene profiling. (**a**) Vertical scatter plot of GFP^+ and RFP^+ percentages in response to overall Bmp4 and Activin A concentrations from Fig. 1d. (**b**) qPCR of analyses of differentiating mouse ESCs. Green dashed box indicate exposure to Bmp4 and gastrulation stage.

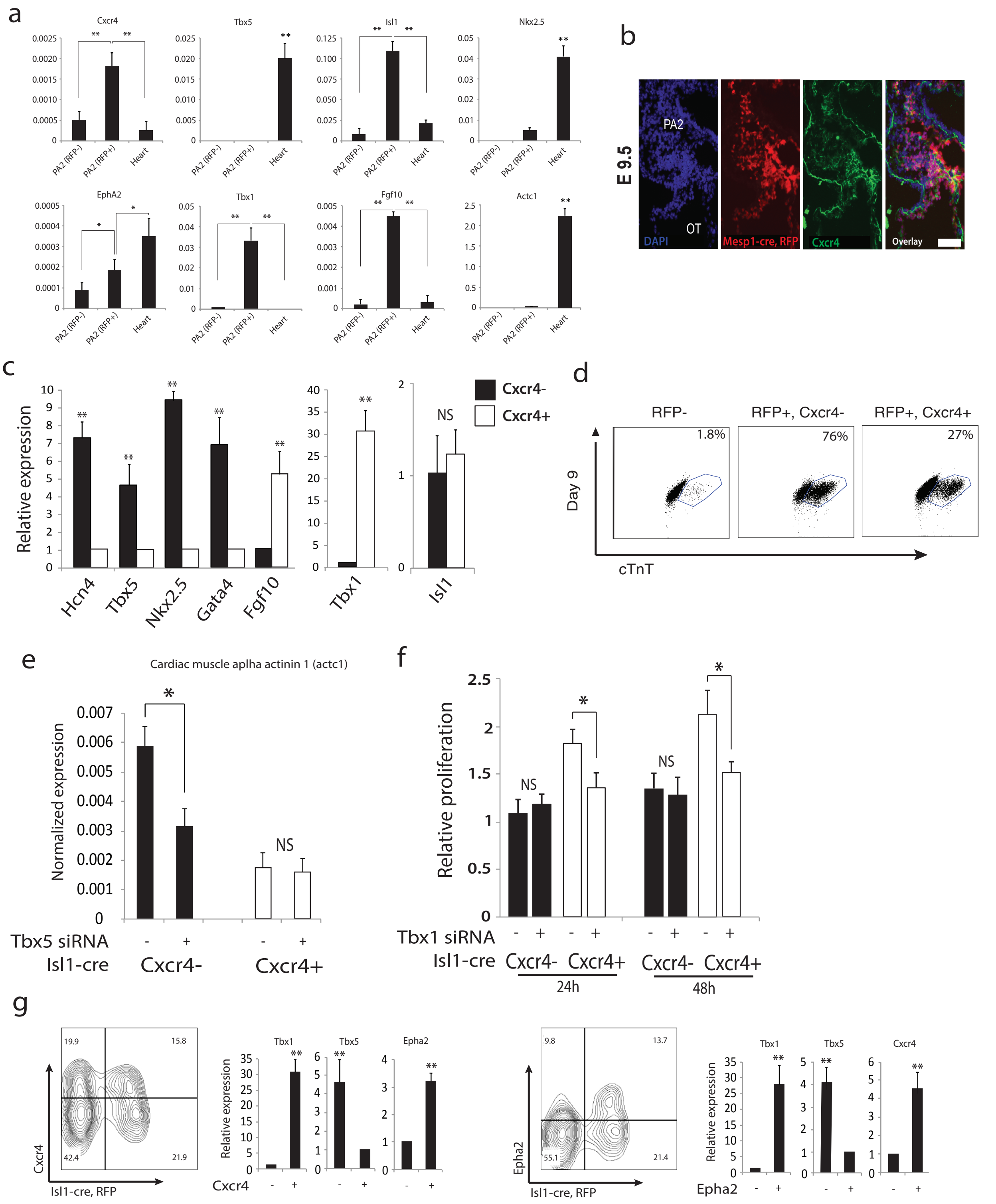

Supplementary Figure 5: Analysis of Cxcr4-positive progenitors in vivo and in vitro. (a) Expression levels of *Cxcr4* and *Epha2* along with *Tbx5, Tbx1, Isl1, Fgf10 Nkx2.5* and *acTc1* in Mesp-cre, RFP$^+$ and RFP$^-$ cells isolated from pharyngeal arches and in developing heart at E9.0. (**b**) Immunohistochemistry analysis of 2nd pharyngeal arch of Mesp1-cre, RFP linage trace analysis (red) and Cxcr4 (green). White scale bars indicate 100µm. (**c**) qPCR analyses of early heart field genes in isolated Isl1-cre, RFP$^+$, Cxcr4$^{-/+}$

cells at day 5.5. (**d**) Representative flow cytometric analyses of RFP-, Isl1-Cre, RFP^+, Cxcr4- and Isl1-Cre, RFP^+, $Cxcr4^+$ cells isolated at day 5.5. (**e**) Cardiac muscle α-actinin 1 (actc1) levels in Isl1-Cre, RFP^+, $Cxcr4^-$/$Cxcr4^+$ derived cells isolated and transfected with siRNA against *tbx5* at day 5.5. Cells were analyzed 3 days after transfection. (**f**) Proliferation analysis of Isl1-Cre, RFP+, $Cxcr4^-$/$Cxcr4^+$ cells 24h and 48h after isolation and transfection with siRNA against *tbx1* at day 5.5. (**g**) Representative flow cytometric analyses of Isl1-Cre, RFP; Cxcr4 and Epha2 cells at day 5.5, and qPCR analyses of Isl1-Cre, RFP^+, $Cxcr4^{+/-}$ and $Epha2^{+/-}$ cells. All data are mean ± SEM; *$p < 0.05$, **$p < 0.01$; $n = 3$; p values were determined using a paired Student's t test.

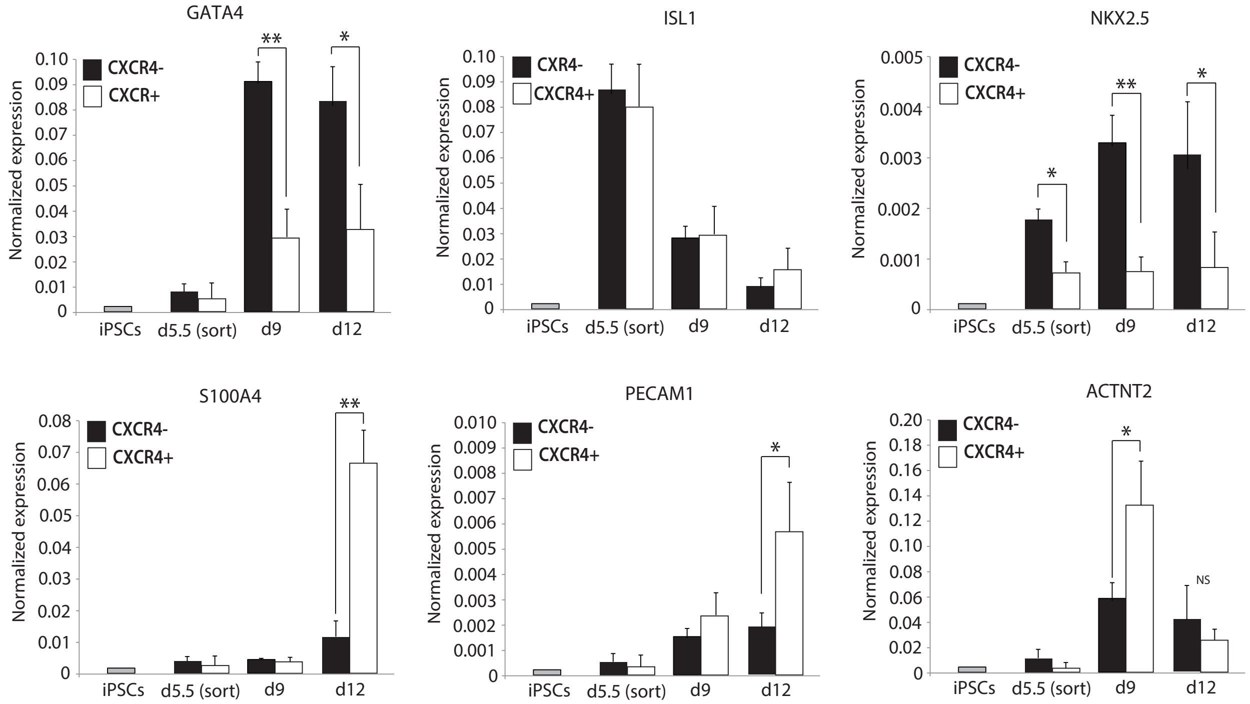

Supplementary Figure 6: qPCR analyses of cardiac lineage markers in hiPSCs and CXCR4+/- cells sorted at day 5.5

All data are mean ± SEM; *p < 0.05; **p < 0.01; n = 3; p values were determined using a paired Student's t test.

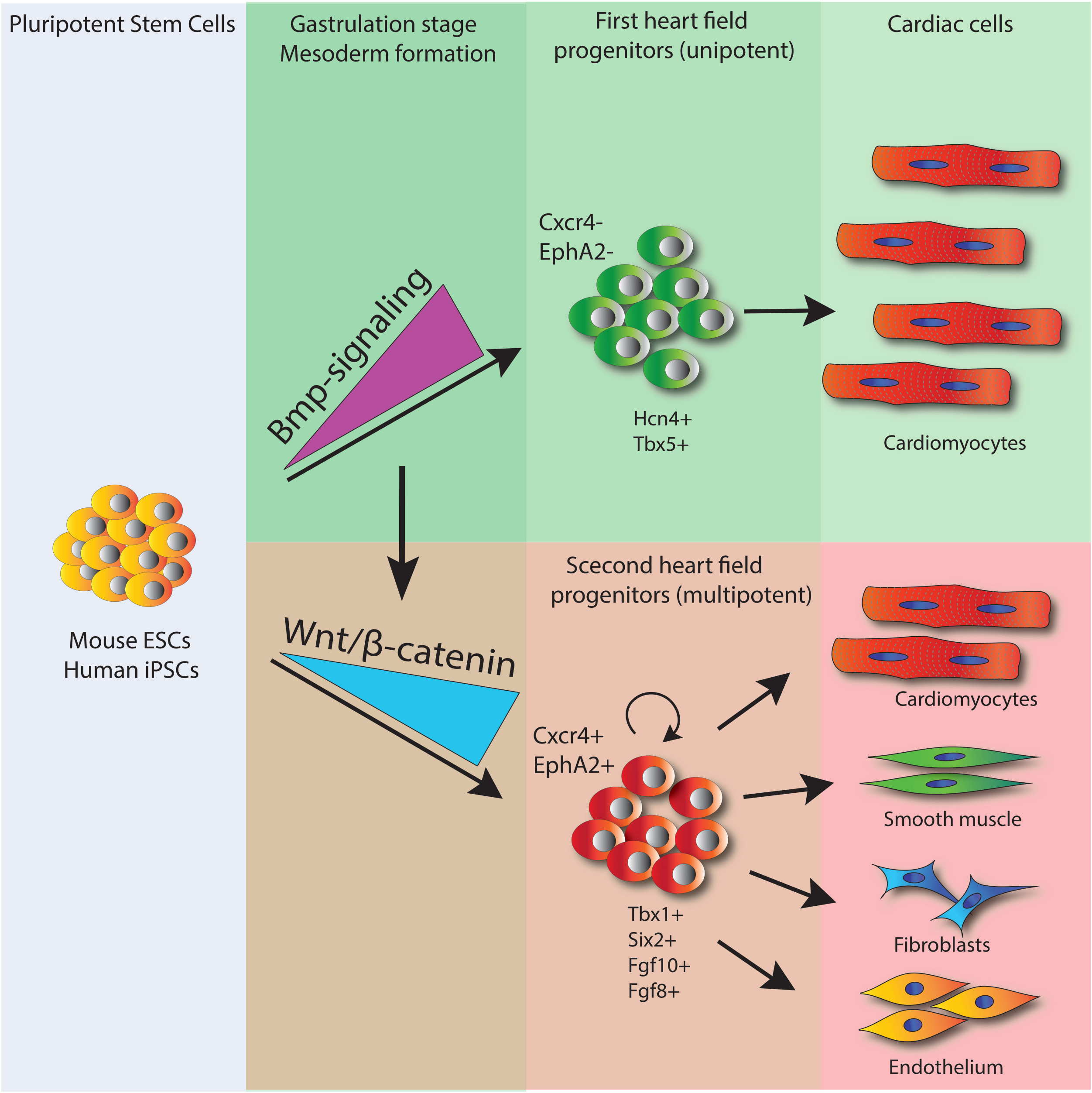

Supplementary Figure 7: Representative scheme of distinct heart field specification during gastrulation by gradients of Bmp and Wnt/β-catenin signaling that can be identified by based on Cxcr4 and EphA2 expression.
