# Supplementary material for: Precardiac organoids form two heart fields via Bmp/Wnt signaling
Source: Nat Commun. 2018 Aug 7;9:3140. doi: 10.1038/s41467-018-05604-8 (PMC6081372; doi:10.1038/s41467-018-05604-8)
Supplement: Supplementary file 2 — Description of Additional Supplementary Files [file 41467_2018_5604_MOESM2_ESM.pdf]

Description of Additional Supplementary Files

File Name: Supplementary Movie 1
Description: Heart field development in a PSC-derived spheroid.

File Name: Supplementary Data 1
Description: RNA-sequencing data.

File Name: Supplementary Data 2
Description: qPCR primer list.

File Name: Supplementary Data 3
Description: Microarray dataset.
